# Focal intratesticular lesions including spontaneous hematomas in the acute scrotum: the pivotal role of ultrasound and MRI in diagnosis and management—a pictorial review

**DOI:** 10.1186/s13244-026-02269-6

**Published:** 2026-04-20

**Authors:** Laurence Rocher, Mariam Hijazi, Fatima Shaito, Chahinez Hani, Omar Bekdache, Emmanuel Arama, Sondes Zaag

**Affiliations:** 1 Department of Radiology, Antoine Béclère Hospital, Paris-Saclay Hospitals, APHP, 157 rue de la porte de Trivaux, 92140 Clamart, France; 2 Paris Saclay University, BIOMAPS, UMR1281, Université Paris Saclay, 63 Rue Gabriel Péri, 94270 Le Kremlin-Bicêtre, France; 3 Department of Radiology, Bicêtre Hospital, Paris-Saclay Hospitals, APHP, 78 Avenue du Général Leclerc, 94275 Le Kremlin Bicêtre, France

**Keywords:** Scrotum, Acute pain, Hematoma, Color doppler ultrasound, Magnetic resonance imaging

## Abstract

**Abstract:**

An acute scrotum with a focal intratesticular lesion represents a diagnostic challenge and may lead to misdiagnosis and inappropriate management, such as unnecessary orchidectomy or empirical antibiotic therapy. In an emergency setting of acute scrotal pain, a wide spectrum of underlying conditions may be encountered, including testicular tumors with necrotic changes, abscesses, infarctions, and spontaneous hematomas. The latter are uncommon and frequently underrecognized entities, carrying a significant risk of mismanagement; therefore, particular emphasis is placed on their imaging features. Multiparametric ultrasound (US), including contrast-enhanced ultrasound and shear wave elastography (SWE), combined with multiparametric enhanced magnetic resonance imaging (MRI), plays a pivotal role in establishing an accurate diagnosis and guiding appropriate treatment decisions. This pictorial review illustrates the broad spectrum of focal intratesticular lesions presenting in the context of acute scrotum, emphasizing the role of imaging in differentiating benign conditions—such as spontaneous hematomas, which can be managed conservatively-from malignant tumors requiring prompt surgery.

**Critical relevance statement:**

Multiparametric ultrasound and MRI improve the diagnostic accuracy of focal intratesticular lesions in acute scrotum, particularly spontaneous hematomas, helping avoid misdiagnosis and unnecessary surgical intervention.

**Key points:**

- Acute scrotum associated with a focal intratesticular lesion is a diagnostic pitfall that may lead to inappropriate management, including unnecessary surgery.
- Multiparametric ultrasound (including CEUS and elastography) and MRI are complementary for distinguishing benign from malignant lesions and guiding management.
- Spontaneous testicular hematomas are rare benign entities characterized by T1-weighted image hyperintensity with a hypointense core and no enhancement on subtraction imaging, supporting conservative treatment.
- Lack of internal enhancement and geographic margins are keys for diagnosing segmental infarction, whereas enhancing thickened or nodular walls and increased stiffness suggest necrotic or hemorrhagic tumors; abscesses typically show dominant inflammatory changes with extra-testicular involvement.

**Graphical Abstract:**

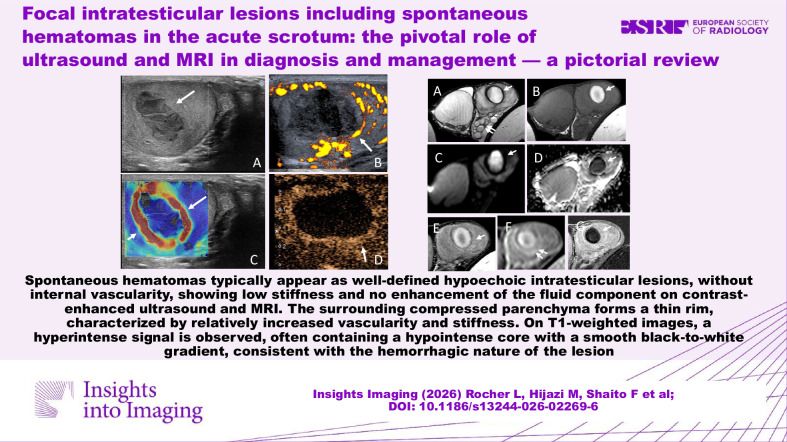

## Introduction

Acute non-traumatic scrotum pain is a common yet challenging issue in the emergency department. While obvious spermatic cord torsion requires urgent surgical intervention without delay, color Doppler ultrasound (US) is also the first-line imaging modality in other situations [1]. It usually allows for the diagnosis of epididymis, orchitis, spermatic cord torsion, or testicular appendage torsion [2]. The detection of one or more intratesticular focal lesions in this setting represents a diagnostic challenge for both the sonographer and the clinician. Indeed, many of these cases are labeled as potential abscess—which are ultimately rare [3]—without confidently excluding a necrotic tumoral process. When orchidectomy is performed, often after weeks of ineffective antibiotic therapy, histopathologic analysis may reveal a benign condition such as hematoma [4] or infarction [5].

As diagnostic percutaneous testicular biopsy remains contraindicated by urological societies if a tumor cannot be confidently excluded [6], the imaging findings play a crucial role in diagnosis assessment. Moreover, surgical management differs according to the suspected diagnosis: a scrotal approach is used for abscess drainage, whereas an inguinal approach is mandatory when malignancy is suspected. Given these considerations, there is a need for accurate imaging to assess the diagnosis before surgical procedures.

With the introduction of contrast-enhanced sonography (CEUS), shear Wave elastography (SWE) and ultrasensitive Doppler (USD), ultrasound has become more specific and is sufficient in the majority of cases to establish the correct diagnosis. However, diagnostic uncertainty may persist in selected cases. In this context, Multiparametric magnetic resonance imaging (MRI) has emerged as a highly accurate diagnostic tool, providing detailed anatomical and functional assessment [7–12]. MRI is particularly valuable as an adjunct to US when findings are inconclusive or discordant with the clinical presentation [4–34]. Compared with the US, MRI is less operator-dependent and offers a wide field of view with multiplanar imaging capabilities, excellent soft-tissue contrast, high sensitivity to contrast enhancement, and additional functional information, allowing accurate characterization of intratesticular and paratesticular lesions.

This pictorial review aims to illustrate the imaging features of rare intratesticular pathologies presenting with acute scrotum, including testicular infarction, abscesses, necrotic tumors and spontaneous hematomas.

### US techniques and MRI protocol

Ultrasound is the first-line imaging modality and basic exam to perform in such cases. A standardized multiparametric US (mpUS) technique is essential to achieve an accurate diagnosis [13, 14]. The patient is positioned supine, and a generous amount of coupling gel should be applied to minimize pressure on the scrotum [15]. A high-frequency linear-array transducer (5–12Mhz) should be used [16]. Imaging parameters must be carefully settled, particularly for Color Doppler US, using a low pulse repetition frequency, to improve the detection of lesion vascularity. When available, USD, such as Angio Planewave Ultrasensitive (Angio-PL.U.S.) or Superb Microvascular Imaging (SMI), are recommended as they significantly improve depiction of small or slow-flow vessels compared with conventional Color Doppler US [17, 18].

Shear wave elastography provides additional diagnostic information. As the testis is a relatively soft organ, with a mean stiffness ranging from 2 to 4 kPa [19], the elastography scale should not exceed 30 kPa to ensure accurate assessment.

To accurately assess lesion vascularization or focal perfusion defects, such as infarction, contrast-enhanced Ultrasonography (CEUS) may be performed following intravenous injection of 4.8 mL of sulfur hexafluoride microbubbles (Sonovue®, Bracco), followed by a flush of saline. Continuous cine-loop acquisition is essential for optimal post-examination analysis [9].

Point-of-care US (POCUS) is increasingly used in emergency settings and is now standard practice in emergency and musculoskeletal medicine. B-mode and Color Doppler are the primary tools used, and their diagnostic performance is actively being evaluated [20–23]. Indeed, when mpUS, including CEUS, is unavailable or inconclusive, MRI provides added diagnostic value for indeterminate lesions.

Scrotal MRI has become an essential second-line modality for the characterization of indeterminate testicular lesions following US [8]. The MRI protocol includes axial, coronal, and sagittal T2-weighted sequences, diffusion-weighted imaging with at least two *b*-values (e.g., 0–800 or 50–1000 s/mm^2^), axial T1-weighted turbo spin-echo sequences, dynamic contrast-enhanced T1-weighted sequences, and post-contrast T1-weighted imaging [24]. In patients with focal lesions, hemorrhagic components may appear hyperintense on unenhanced T1-weighted images. Subtraction imaging between pre- and post-contrast T1-weighted sequences is therefore essential to determine the presence or absence of lesion enhancement. All sequences are acquired without fat suppression.

### Clinical and imaging data

From 2013 to 2025, a query of the Paris-Saclay hospitals' databases identified several patients presenting with acute scrotum and focal intratesticular lesions, including abscesses, infarctions, spontaneous hematomas, and necrotic tumors. Table 1 summarizes the main US and MRI features associated with each main diagnosis.

**Table 1:** US and MRI findings of the differential diagnosis of acute scrotum with intratesticular lesion

	Clinical findings	US				MRI					
		B Mode	Color doppler	CEUS	SWE	T1-weighted image	T2-weighted image	Diffusion	ADC	Dynamic enhanced	T1 substarction
Spontaneous hematoma	Acute pain and swelling without fever	Ovoid hypoechoic lesion with rapid changes during the first weeks/if multiple: similar findings/no microlihts/normal epididymis	Absence of vascularization of the content/hypervascularization outside the lesion	Absence of enhancement of the content/enhancement of a thin rim outside the lesion	Low stiffness of the content/high stiffness of the compressed peripheral parenchyma	Dominant hypersignal, a central core in hyposignal	Hypersignal	Hypersignal compared to the testis	Low ADC value compared to the testis	Slight hyperenhancement outside the lesion	No enhancement of the content
Abcess	Acute pain/fever	Epididymal swelling Hypoechoic area, fluid content	Absence of vascularization of the content/hypervascularization of the epididymis and the parenchyma	Absence of enhancement of the content/enhancement of a thin rim outside the lesion	Low stiffness of the content/high stiffness of the compressed peripheral parenchyma	Isosignal	Intermediate hypersignal	Hypersignal compared to the testis	Low ADC value compared to the testis	Hyperenhancement of the testis outside the abcess	No enhancement of the content
Infarction	Acute pain without fever	Normal for the first hours, then hypoechoic area	Absence of vascularization of the lesion, with hypervascularization of the borders	Defect of enhancement with geographic pattern and hypervascularized border	Increased stiffness of the periphery	Dominant isosignal, some part show some hypersignal on T1 in case of hemorrhagic transformation	Intermediate hypersignal, with border in hyposignal. May be heterogeneous	Mixed hyper and hyposignal	Mixed high ADC value of the necrosed portion and low ADC value of the borders	Absence of enhancement of the infarcted area with geographical pattern	Absence of enhancement of the infarcted area
Necrosed tumor	Acute pain without fever	Hypoechoic lesion, irregular rim/microliths/other nodules	Vessels in the solid part	Enhancement of the solid part, with potential nodularity	Increased stiffness of the lesion	Dominant isosignal, some part showing some hypersignal on T1 in case of hemorraghic transformation	Heterogeneous signal	Hypersignal of the solid peripheral part	Low ADC value of the solid peripheral part	Enhancement od the solid peripheral part including the nodularity if present	Enhancement of the solid peripheral part and nodularities

*US* ultrasound, *MRI* magnetic resonance imaging, *CEUS* contrast-enhanced ultrasound, *SWE* shear wave elastography, *ADC* apparent diffusion coefficient

#### Spontaneous hematomas

This rare condition, initially described in 1914 as idiopathic intratesticular hemorrhage [25], has since been reported only sporadically. In most published cases, the final diagnosis was established after orchiectomy. Reported cases are summarized in Table 2 [4, 25–36].

**Table 2:** Review of cases on spontaneous hematomas

Cases (Ref)	Year	Patients number	Medical history	Side	Age (years)	US	CEUS	MRI	Surgery	Follow up
Barrington FJF [25]	1914	1	None	R	24	No	No	No	Orchidectomy	
Altaffer LF [33]	1980	2	None	NA	NA	No	No	No	Orchidectomy	
Evans KJ [32]	1985	1	None	L	28	No	No	No	Orchidectomy	
Ovesen P [31]	1991	1	None	L	25	Yes	No	No	Evacuation	NA
Sinclair J [30]	2003	1	None	R	16	Yes	No	No	Evacuation	NA
Yuksel MB [36]	2011	1	None	L	15	No	No	No	Orchidectomy	
Gaur S [29]	2011	1	None	R	40	Yes	No	Yes, 84 days after the pain	Orchidectomy	
Takeuchi T [28]	2017	1	None	L	21	No	No	No	Orchidectomy	
Mathur M [35]	2017	1	None	R	NA	Yes	No	Yes	NA	NA
Bertolotto M [34]	2018	1	None	L	NA	Yes	Yes	Yes (not shown)	NA	NA
Xue N [27]	2022	1	None	NA	NA	Yes	Yes	No	Orchidectomy	
Abuorouq S [4]	2023	1	None	L	15	Yes	No	No	Orchidectomy	
Jhang JJ [26]	2023	1	Reiter’s syndrome	R	15	Yes: initial vascularized nodule	0	Yes, 12 weeks after	no	NA

All reported patients share a similar clinical presentation, characterized by acute scrotal pain and swelling without fever. Hematomas may be multiple within a single testis and can be bilateral. This entity is likely underestimated, as some cases are ultimately attributed to minor or forgotten trauma [37]. The primary cause of such hemorrhage is not well understood, except in one case attributed to sulfasalazine in Reiter’s syndrome [26]. The pathological analysis found interstitial hemorrhage while seminiferous tubules were intact, and assessed that those results differed from pathological findings after hemorrhage in the case of spermatic cord torsion [36].

**Fig. 1:**
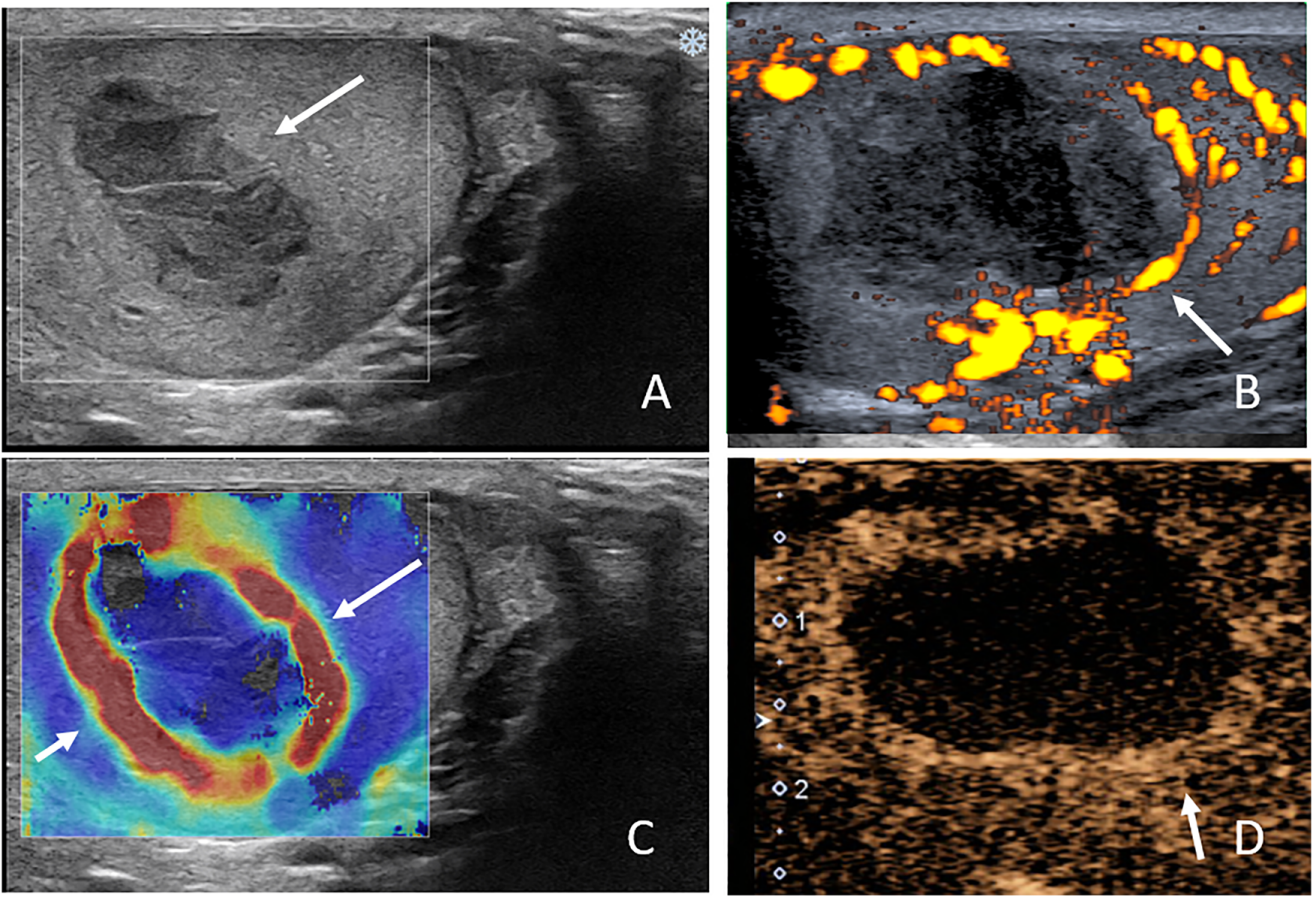
Multiparametric US performed 21 days after symptom onset in a 44-year-old patient with histologically proven spontaneous intratesticular hemorrhage. Despite the supposed diagnosis, urologists prompted an orchidectomy. **A** B-mode US showing an ovoid hypoechoic lesion with internal septations (initial US not shown demonstrated echogenic content). **B** Power Doppler US showing absence of internal vascularity, with increased vascularity of the peripheral rim and adjacent parenchyma. **C** Shear wave elastography demonstrating low stiffness of the lesion (2.7 kPa) and increased stiffness of the regular peripheral rim (61.8 kPa). **D** Contrast-enhanced US showing absence of internal enhancement and peripheral rim hyperenhancement

**Fig. 2:**
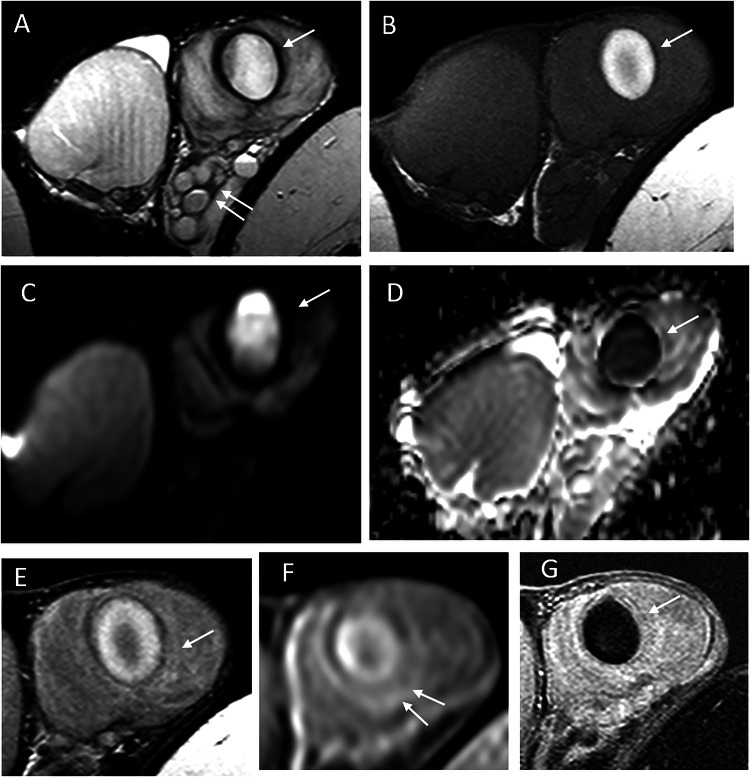
Scrotal MRI of the same patient, performed 28 days after symptom onset, is consistent with a late subacute hematoma. **A** T2-weighted image showing a hyperintense lesion with a hypointense rim (arrow), consistent with hemosiderin deposition; dilated scrotal veins are visible (double arrows). A striated pattern of the adjacent parenchyma is noted. **B** Predominant hyperintensity on T1-weighted images, with areas of lower signal intensity corresponding to extracellular methemoglobin. **C** Diffusion-weighted imaging (high b-value) showing hyperintensity, with corresponding hypointensity on the ADC map (**D**). **E** Post-contrast T1-weighted image showing a striated enhancement pattern of the parenchyma, making assessment of lesion enhancement difficult. **F** Early dynamic contrast-enhanced sequence showing slightly increased enhancement of the adjacent parenchyma. **G** Subtraction image confirming the absence of internal lesion enhancement

**Fig. 3:**
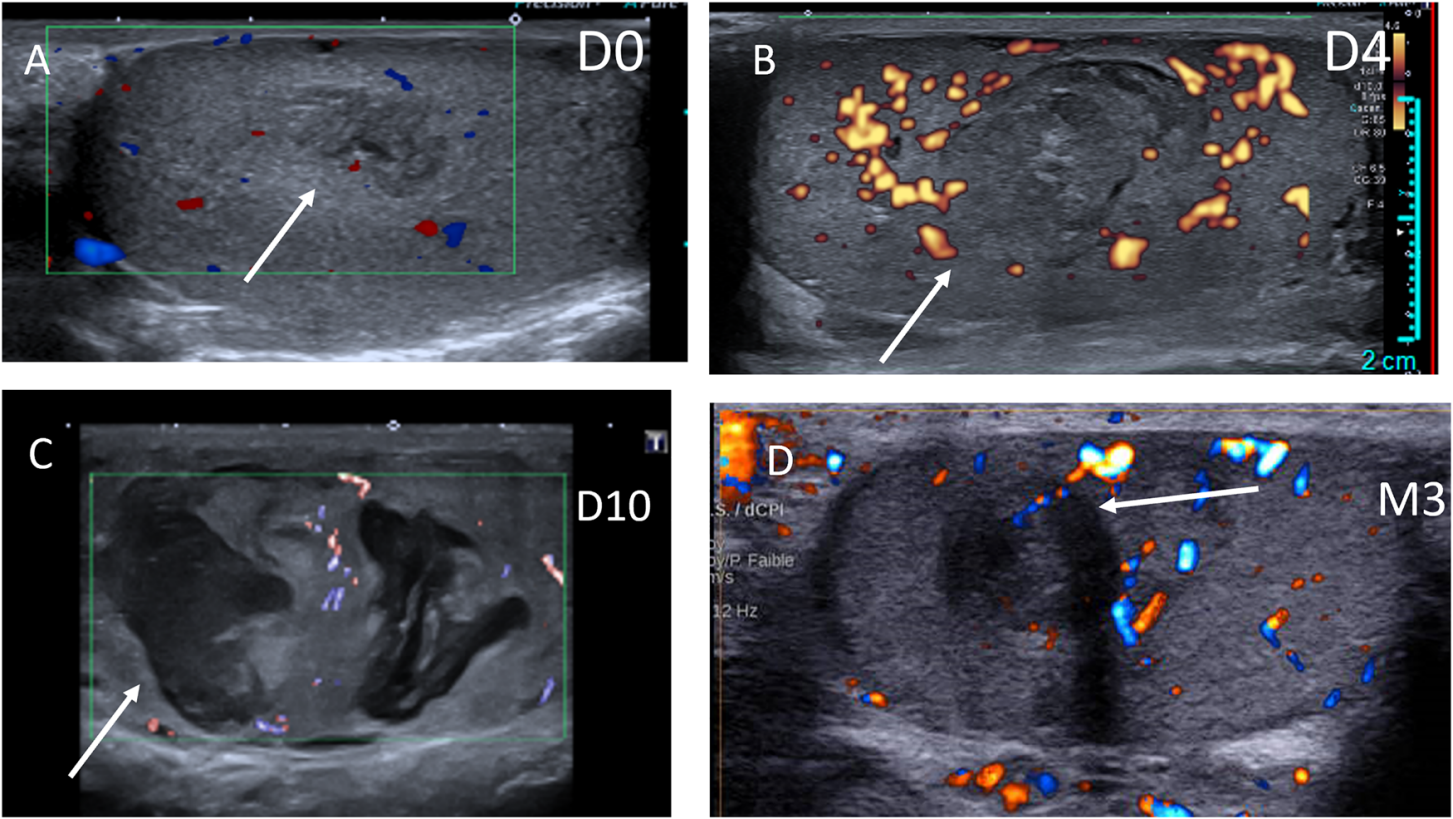
Bilateral spontaneous intratesticular hematomas in a 50-year-old man with liver cirrhosis; only the right testis is shown. **A** Day 0: small hypoechoic avascular lesion. **B** Day 4: follow-up US performed due to increased pain shows a larger, heterogeneous, echogenic avascular lesion. **C** Day 10: partial liquefaction of the hematoma. **D** Month 3: partial regression with persistence of a hypoechoic vascularized lesion mimicking a tumor. The patient remains under follow-up for cirrhosis, with an intact scrotum 4 years later

**Fig. 4:**
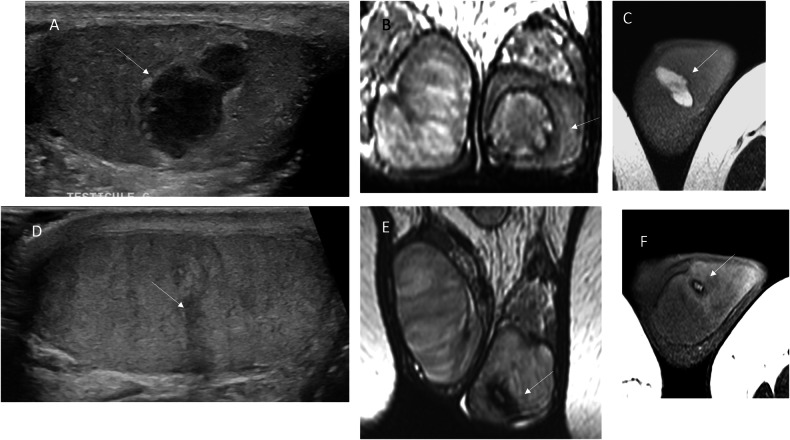
Spontaneous hematoma in a 38-year-old patient. Initial US and MRI were performed 4 days after symptom onset, with 6-week follow-up imaging. **A** (US), **B** (MRI, T2-weighted sequence) and **C** (MRI, T1-weighted sequence): Initial examinations showing features similar to other cases, with additional striated heterogeneous signal of the surrounding parenchyma. **D** (US) Follow-up US showing regression of the hematoma, leaving a small hypoechoic scar (arrow). **E** (MRI, T2-weighted sequence) and **F** (MRI, T1-weighted sequence): residual lesion with a marked hypointense rim consistent with hemosiderin deposition

**Fig. 5:**
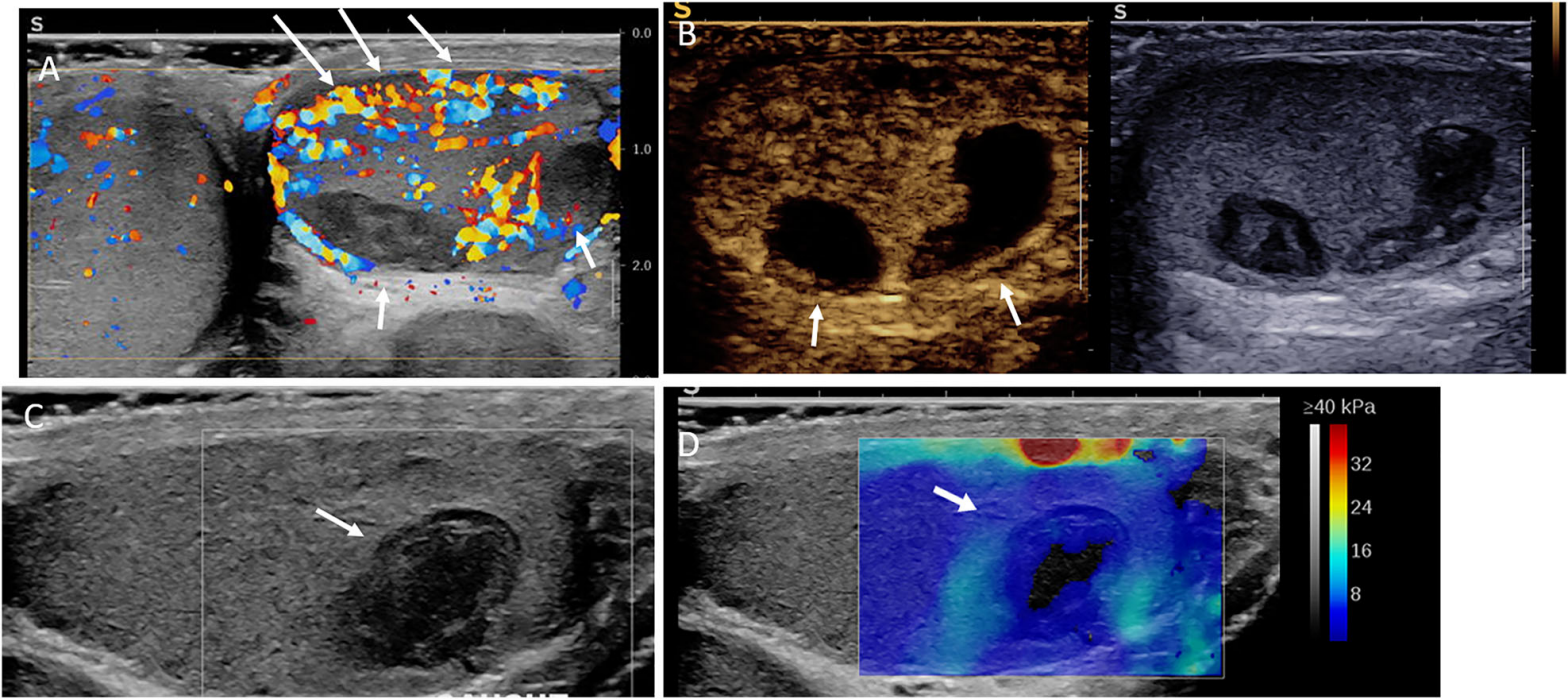
US findings performed 10 days after symptom onset in a 34-year-old man with multiple spontaneous hematomas. **A** Increased vascularity of the surrounding parenchyma (arrows). **B** Absence of enhancement on CEUS. **C**,** D** B-mode US and SWE showing absence of central stiffness and a slight increase in stiffness of the adjacent parenchyma

**Fig. 6:**
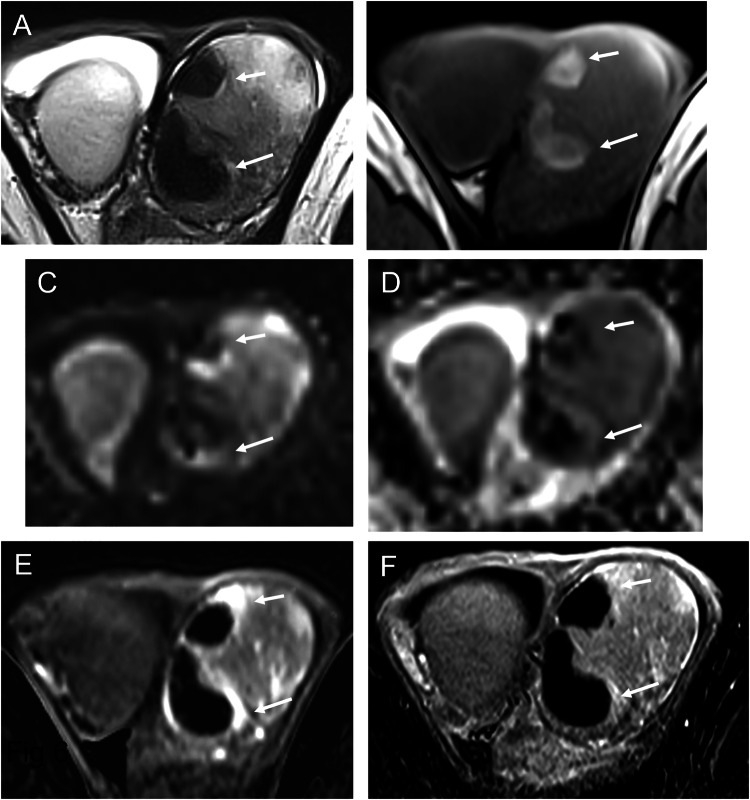
MRI findings of the previous case (Fig. 5) performed 10 days after symptom onset. These findings illustrate signal variability related to early imaging, with hematomas appearing hypointense on T2-weighted images (**A**) and partially hyperintense on T1-weighted images (**B**), reflecting the presence of both intracellular and extracellular methemoglobin. Diffusion-weighted imaging (**C**,** D**) is affected accordingly, whereas T1-weighted imaging and subtraction sequences after gadolinium injection (**E**,** F**) are helpful for lesion characterization

**Fig. 7:**
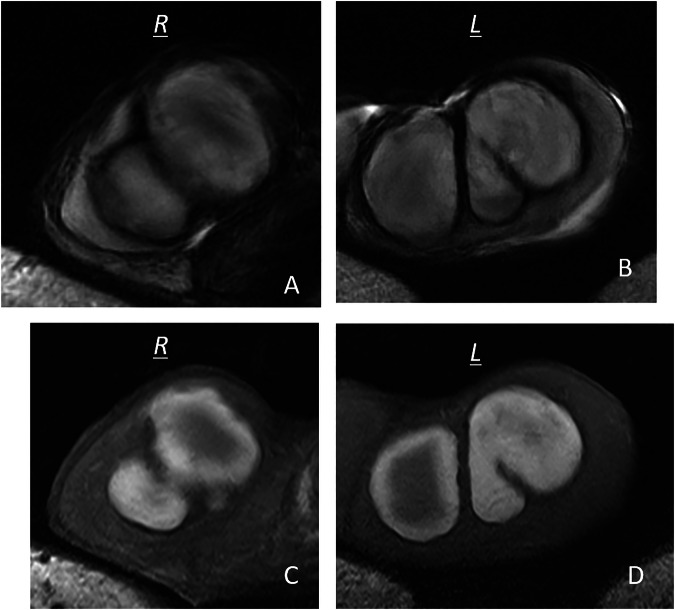
Bilateral spontaneous hematomas in a patient with liver cirrhosis, MRI findings (same patient as in Fig. 3). Typical hematomas as previously described, with rims in hypo signal on T2-weighted images (**A**,** B**) and exhibiting a hypersignal including an internal core in isosignal on T1-weighted images (**C**,** D**). Additional sequences could not be acquired due to patient discomfort

On US, spontaneous hematomas typically appear as hypoechoic, avascular lesions that may be ovoid or crescent-shaped. A thin peripheral rim of increased vascularity is frequently observed on color Doppler US, ultrasensitive Doppler, or CEUS. SWE demonstrates low stiffness within the lesion and increased stiffness of the peripheral rim. Follow-up imaging usually shows progressive liquefaction and size reduction, eventually resulting in a scar-like appearance Figs. 1 to 7).

MRI plays a key role in confirming the hemorrhagic nature of these lesions. Typical findings include a thin hypointense outer rim on both T1- and T2-weighted images, hyperintense signal on T1-weighted images corresponding to methemoglobin, and a central core with isointense signal and a smooth signal gradient. On T2-weighted images, the lesion content is usually hyperintense. Diffusion-weighted imaging shows high signal intensity on high b-values with corresponding low signal on ADC maps. After gadolinium administration, no internal enhancement is observed on subtraction images, whereas the compressed adjacent parenchyma may show increased enhancement on dynamic sequences (Figs. 2, 4, 6, 7). Signal characteristics may vary depending on the timing of the MRI relative to symptom onset. Early imaging may show signal intensity similar to normal testicular parenchyma on T1-weighted images and low signal on T2-weighted images, reflecting early stages of hemoglobin degradation (Fig. 6). Overall, signal evolution follows patterns similar to those described for intracranial hematomas.

Given the predominantly left-sided involvement, the relatively young age of affected patients, and the frequent visualization of dilated scrotal veins, venous hypertension or varicocele may represent a predisposing factor. This mechanism could contribute to vascular rupture and interstitial bleeding, as described in histopathological analyses [36].

#### Abscesses

Testicular abscesses are uncommon compared with epididymitis, epididymal abscesses, or orchitis [38, 39]. In bacterial testicular infections, pain typically increases progressively and decreases gradually with antibiotic therapy. Testicular abscesses are usually associated with epididymitis and inflammatory involvement of extra-esticular tissues and have also been described following urological catheterization [40]. Once a purulent collection has formed, antibiotic therapy alone is often insufficient, and surgical intervention is frequently required. A testis-sparing approach using a scrotal incision may be attempted to preserve testicular tissue [38]. However, testicular loss remains a common outcome due to necrosis or ischemia [39, 41–44]. This diagnosis may also be difficult, and unnecessary orchidectomies are described [45]. In contrast, scrotal tuberculosis usually presents with subacute or chronic symptoms and palpable nodular or bead-like masses [46].

On US, both abscesses and hematomas may appear as hypoechoic, pseudo-solid, avascular lesions on color Doppler imaging, often associated with surrounding intratesticular hyperemia. CEUS typically demonstrates peripheral rim enhancement without internal contrast uptake [47, 48]. However, based on US findings alone, abscesses cannot always be reliably differentiated from spontaneous hematomas, infarctions, or necrotic tumors [49, 50].

**Fig. 8:**
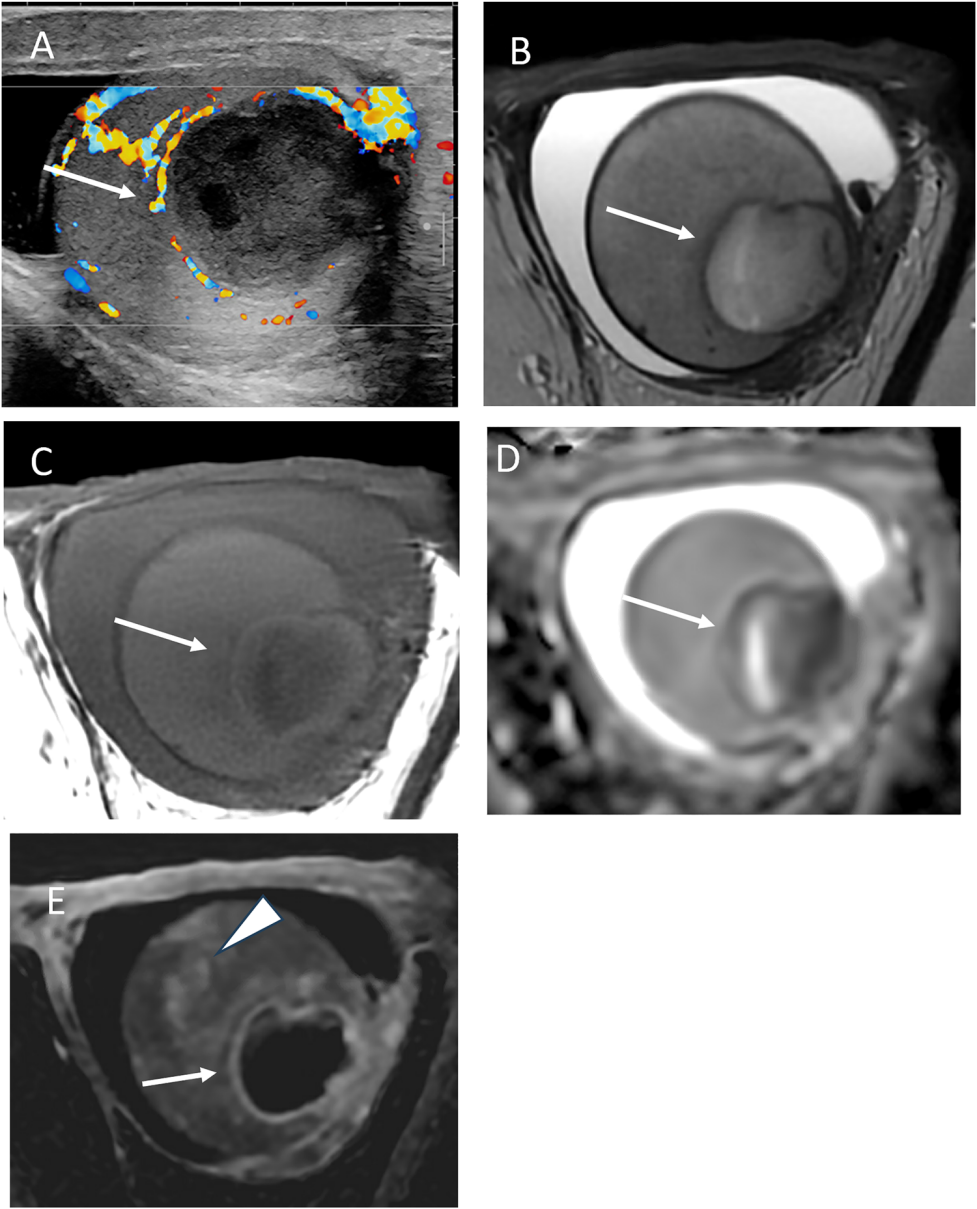
Syphilitic gumma in a patient with confirmed tertiary syphilis. Regression after appropriate antibiotherapy. **A** B-mode US coupled with Colour Doppler showing a heterogeneous lesion and absence of vascularity. **B** T2-weighted MRI showing mixed hyperintense and isointense signal. **C** T1-weighted MRI showing a very thin rim without clear hyperintensity. **D** ADC map showing heterogeneous signal. **E** Post-contrast T1-weighted MRI showing thin rim enhancement (long arrow) with small enhanced foci (arrowhead) and no internal enhancement

**Fig. 9:**
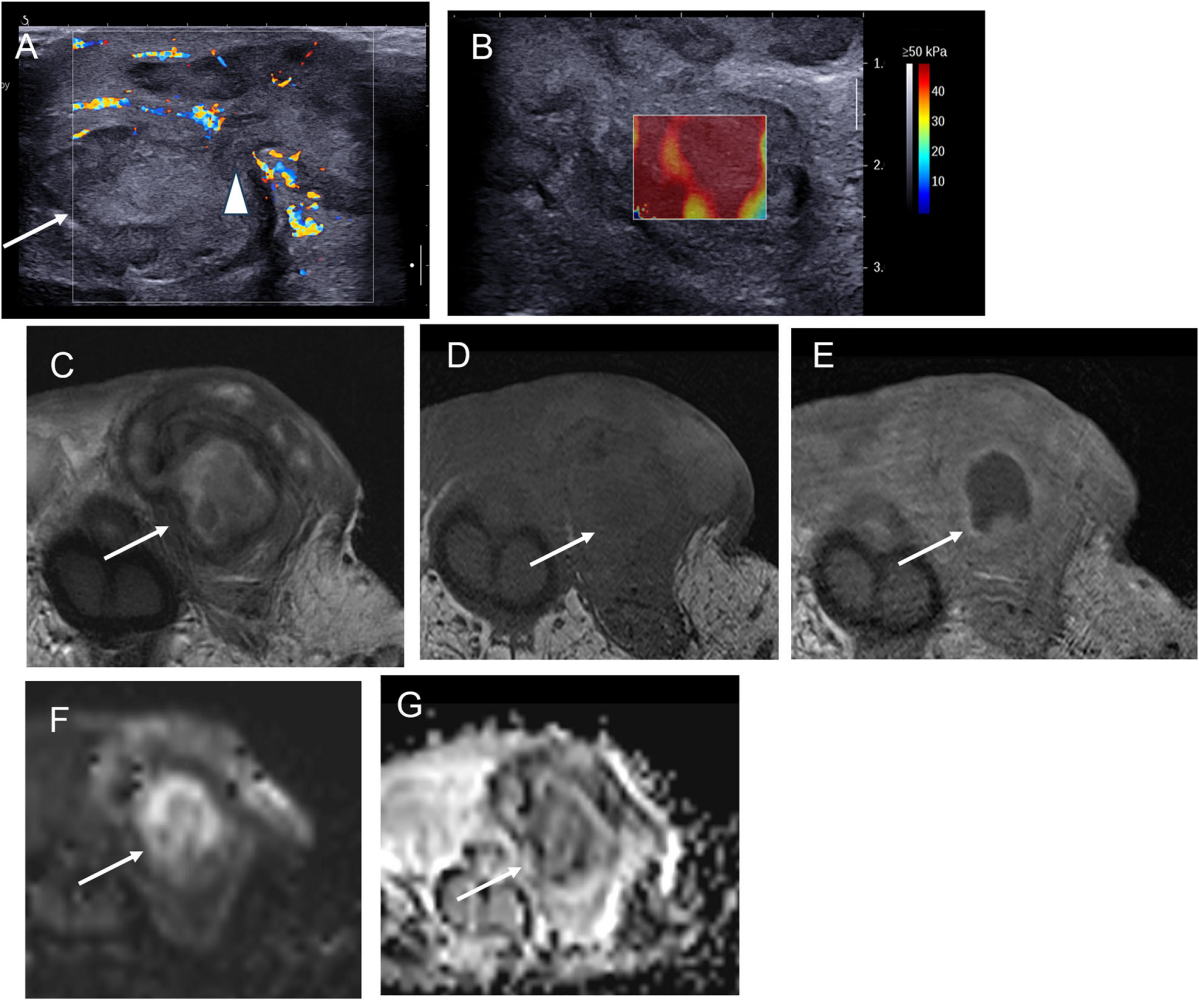
Adverse outcome of bacterial testicular infection with testicular necrosis following severe epididymo-orchitis. **A** Absence of vascularity on Doppler US (arrow), with fistulization (arrowhead). **B** Increased stiffness on SWE. **C** Predominant hyperintensity on T2-weighted MRI. **D** Absence of hyperintensity on T1-weighted image. **E** Peripheral rim enhancement (arrow). **F**,** G** Restricted diffusion on high b-value images and ADC map

**Fig. 10:**
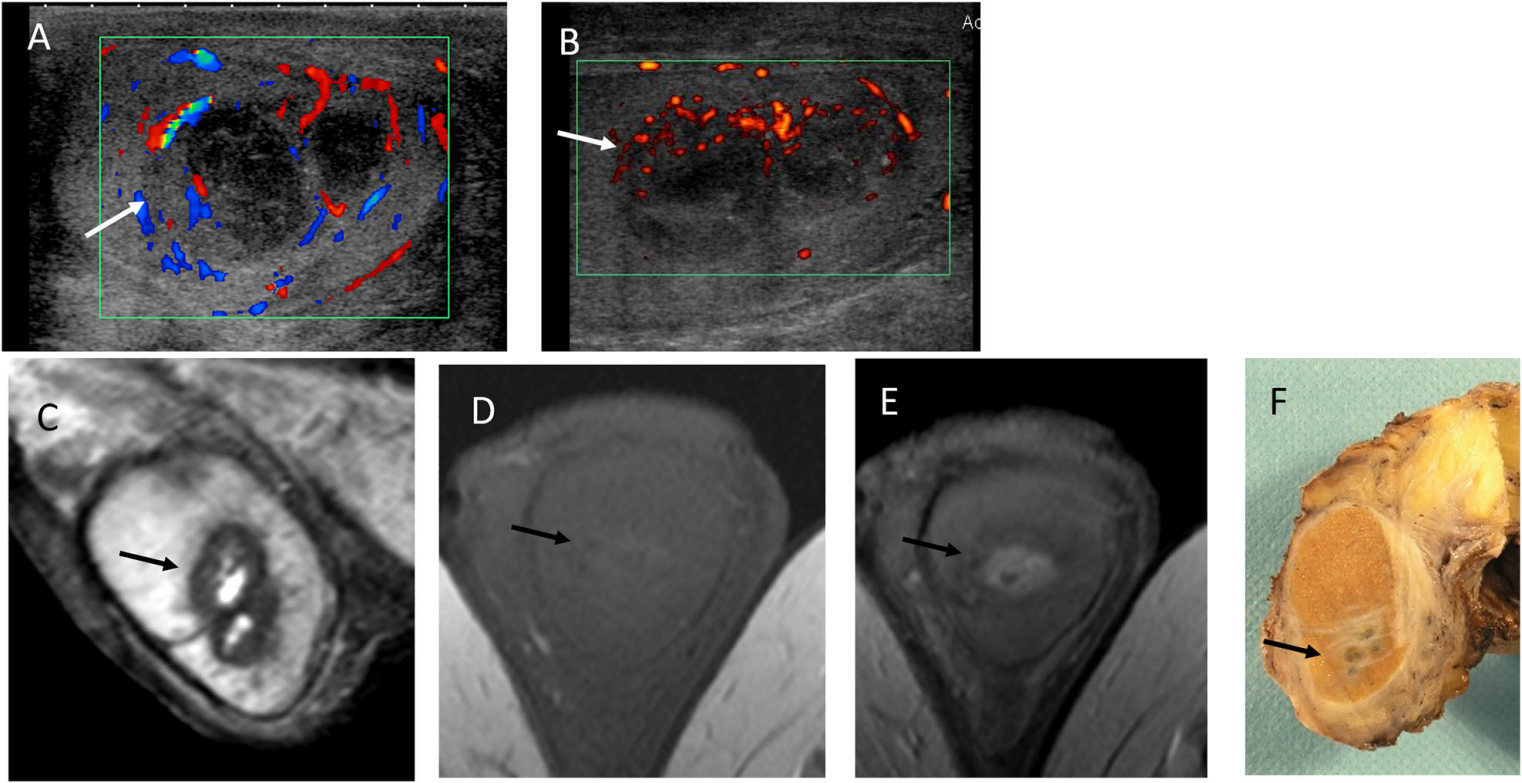
Histologically proven testicular abscesses. **A** Two hypoechoic heterogeneous avascular lesions on color Doppler US. **B** Partial regression after antibiotic therapy. **C** MRI showing a hyperintense core on T2-weighted images with a thick hypointense rim. **D** No marked hyperintensity on T1-weighted images. **E** Thickened peripheral rim enhancement after contrast administration. **F** Macroscopic specimen following orchiectomy, confirming regressive abscesses

The MRI literature on testicular abscesses is limited, mainly describing cases related to tuberculosis [51] or brucellosis infections [52], which may not reflect imaging features of more common bacterial infections. Some case reports, case series, or pictorial reviews described extra-testicular abscesses or complications [53, 54]. At MRI, the signal of the lesion in the T1-weighted image is hypo or isosignal (and not hyper signal as hematomas). MRI findings typically include hypointense or isointense signal on T1-weighted images, marked diffusion restriction with low ADC values, and peripheral rim enhancement. Representative cases illustrating US and MRI findings are shown in Figs. 8–10.

#### Infarction

**Fig. 11:**
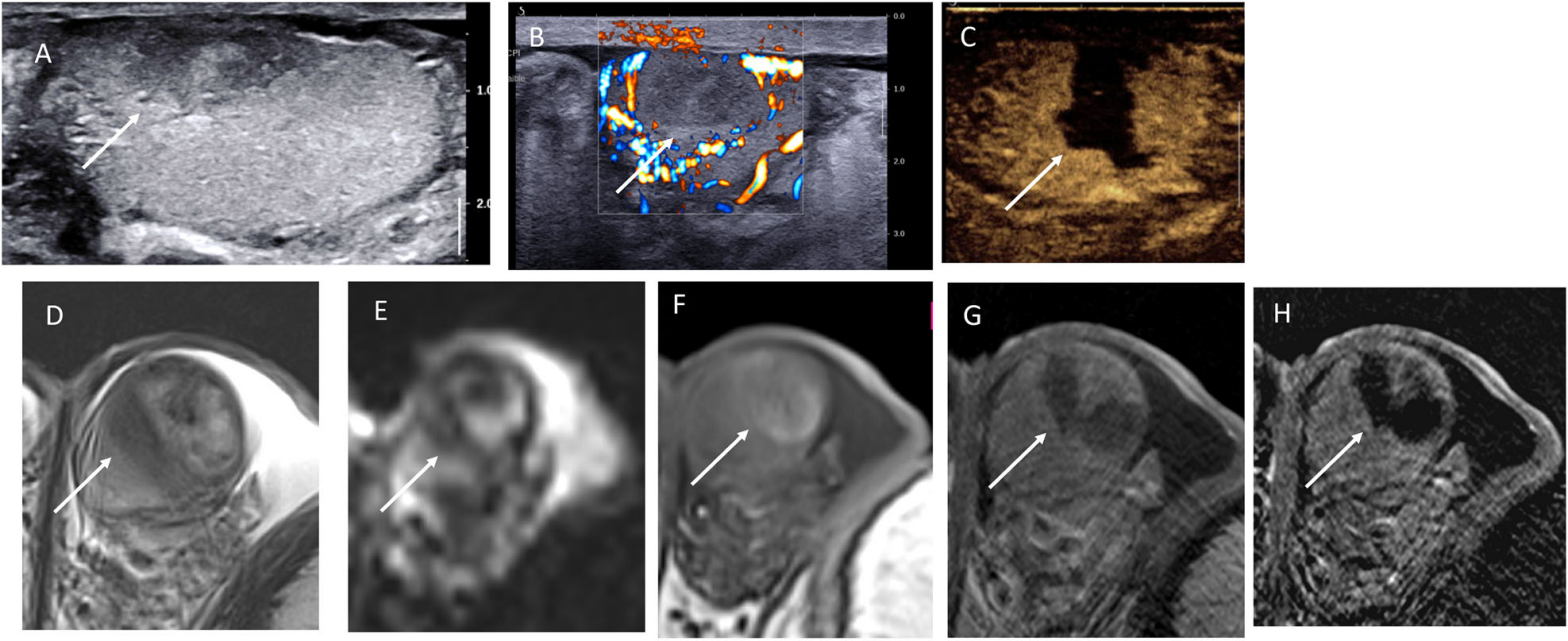
Segmental testicular infarction: US and MRI performed 48 hours after sudden-onset pain. **A** Hypoechoic lesion on B-mode US. **B** Peripheral hypervascularity on ultrasensitive Doppler US. **C** CEUS showing absence of contrast enhancement within a wedge-shaped lesion. **D** T2-weighted MRI showing a wedge-shaped hypointense lesion with well-defined borders. **E** ADC map showing diffusion restriction at the periphery. **F** T1-weighted MRI showing focal hyperintensity consistent with partial hemorrhagic transformation. **G** Post-contrast T1-weighted MRI showing absence of enhancement. **H** Subtraction image confirming lack of enhancement

Testicular infarction is another cause of acute, sudden scrotal pain and may present as a focal intratesticular lesion. It results from occlusion of a small intratesticular artery, leading to localized ischemia and necrosis. Most of them are idiopathic [55] without vascular risk factors, although associations have been reported with several diseases, including Protein C deficiency [56], Sickle cell disease [57], vasculitis [58], SARS-Cov-2 infection/diabetic vasculopathy [59] or arterial atheroma [5]. The US may be normal in the early phase. A hypoechoic area typically becomes visible on B-mode imaging after 1 or 2 days [60]. Color Doppler and ultrasensitive Doppler demonstrate an avascular area with peripheral hyperemia, best assessed using CEUS, which highlights the geographic configuration of the lesion during arterial, venous, and late phases [5, 34, 61, 62]. MRI can also confirm the diagnosis. Infarctions typically demonstrate a wedge-shaped configuration, helping to differentiate them from hematomas, although hemorrhagic transformation may result in focal T1-weighted-image hyperintensity (Fig. 11). Signal distribution remains distinct from spontaneous hematomas, with a thick hypointense rim on T2-weighted images. Diffusion-weighted imaging shows restricted diffusion within the infarcted area [63–65]. Both CEUS and MRI are valuable for diagnosis, although several published cases could not benefit from a conservative approach, and orchidectomies are still performed [66].

#### Tumors with acute necrosis

Testicular cancer usually presents as a painless testicular mass or incidental finding on US (infertility screening) [67]. On US, seminomatous germ cell tumors typically appear as well-circumscribed, homogeneously hypoechoic masses, whereas non-seminomatous germ cell tumors (NSGCT) are more heterogeneous, often containing areas of necrosis, hemorrhage, fibrosis, cystic change, or calcifications [68]. Tumor vascularity is commonly used to differentiate malignant from benign lesions, although some tumors may appear poorly vascularized due to fibrosis (burned-out tumors), necrosis, or hemorrhagic transformation [69]. The diagnosis of testicular cancer is typically established following orchidectomy and subsequent pathological examination [67]. Elastography and contrast-enhanced US may be helpful to differentiate malignant from benign lesions, where malignant lesions usually show increased stiffness in the tumor compared with the normal testis [70, 71]. MRI is increasingly used to discriminate between benign and malignant intratesticular lesions [8].

When tumors present in the context of acute scrotal pain, lesions frequently exhibit hemorrhage or acute necrosis and may be relatively large [72–74]. Rarely, it may result from torsion of the spermatic cord secondary to a testicular mass. Although the US usually establishes the diagnosis by demonstrating vascularized solid components, interpretation may be challenging when solid tissue is limited or absent, complicating both US and MRI assessment [72].

**Fig. 12:**
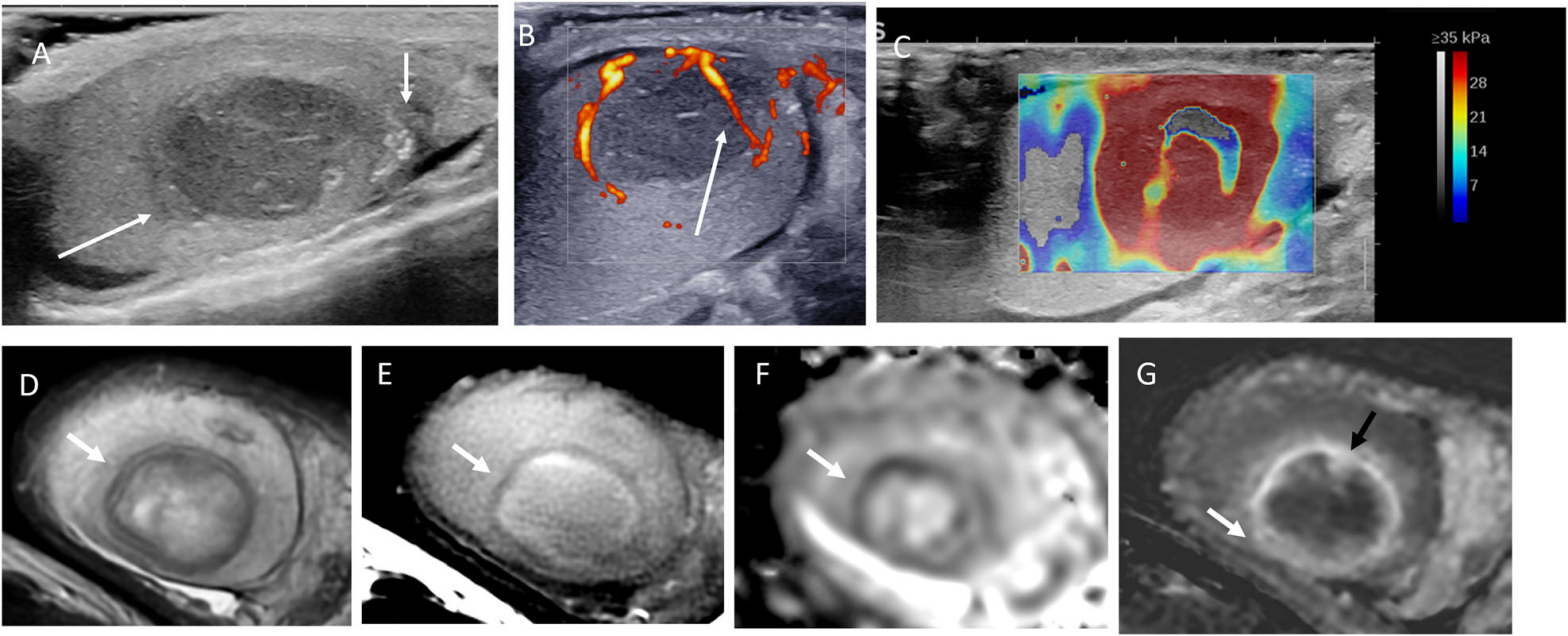
Hemorrhagic and necrotic malignant germ cell tumor presenting with acute scrotal pain. **A** B-mode US showing an irregular, thickened peripheral rim (long arrow) and clustered microliths (short arrow). **B** Power Doppler US showing a small peripheral and intralesional vessel. **C** SWE is demonstrating high stiffness of both the peripheral rim and central components. **D** T2-weighted MRI image showing a thick-walled lesion with heterogeneous signal intensity. **E** T1-weighted MRI image showing subtle peripheral hyperintensity suggestive of hemorrhage. **F** ADC map demonstrating low ADC values within the lesion wall. **G** Post-contrast T1-weighted MRI image with subtraction showing irregular enhancement of the wall (white arrow) and focal internal enhancement (black arrow)

We present a case of NSGT revealed by acute pain, with necrosis and hemorrhagic transformation. The thickened wall appeared hypointense on T2-weighted images, T1-weighted images, and ADC maps, which can mimic the others diagnosis. Subtle irregular nodular enhancement on MRI and USD, along with clustered microliths, contributed to assessing the diagnosis and illustrating the complementarity of both techniques (Fig. 12).

## Conclusion

Multiparametric US combined with MRI may help clinicians establish the correct diagnosis in patients presenting with an acute scrotum and focal intratesticular lesions. Accurate imaging assessment can help avoid inappropriate orchiectomy in cases of abscesses, testicular infarctions, and spontaneous hematomas, while appropriately supporting surgical management when malignancy is suspected.

## Data Availability

Data and materials are available.

## Abbreviations

CEUS: Contrast-enhanced ultrasound
MRI: Magnetic resonance imaging
SWE: Shear wave Elastography
US: Ultrasound
USD: Ultrasensitive doppler

## Declarations

### Study design

Retrospective.
